# RNA Methylation in ncRNA: Classes, Detection, and Molecular Associations

**DOI:** 10.3389/fgene.2018.00243

**Published:** 2018-07-12

**Authors:** Giulia Romano, Dario Veneziano, Giovanni Nigita, Serge P. Nana-Sinkam

**Affiliations:** ^1^Internal Medicine “Division of Pulmonary and Critical Care Medicine”, Virginia Commonwealth University Health System, Richmond, VA, United States; ^2^Department of Cancer Biology and Genetics, The Ohio State University, Columbus, OH, United States

**Keywords:** RNA, methylation, epigenetics, non-coding RNAs, RNA methodologies

## Abstract

Nearly all classes of coding and non-coding RNA undergo post-transcriptional modification, as more than 150 distinct modification types have been reported. Since RNA modifications were first described over 50 years ago, our understanding of their functional relevance in cellular control mechanisms and phenotypes has truly progressed only in the last 15 years due to advancements in detection and experimental techniques. Specifically, the phenomenon of RNA methylation in the context of ncRNA has emerged as a novel process in the arena of epitranscriptomics. Methylated ncRNA molecules may indeed contribute to a potentially vast functional panorama, from regulation of post-transcriptional gene expression to adaptive cellular responses. Recent discoveries have uncovered novel dynamic mechanisms and new layers of complexity, paving the way to a greater understanding of the role of such phenomena within the broader molecular cellular context of human disease.

## Introduction

Up until recently, the central dogma (Crick, 1970) had supported primary focus on the molecular contributions of DNA and protein to human disease. The inability to detect and evaluate RNA with the necessary molecular resolution and precision has limited our understanding of the spectrum of RNA modifications that may drive disease.

Following the discovery of *pseudouridine* (Davis and Allen, 1957), nine additional modifications were identified in 1965 (Holley et al., 1965b). Finally, modification events in nucleotides of mRNA molecules were also uncovered in the 1970s (Desrosiers et al., 1974; Adams and Cory, 1975; Dubin and Taylor, 1975; Perry et al., 1975). Gradually, the “static” interpretation of the cellular role of RNA started to be challenged (Gilbert, 1986). With the discovery of novel species of non-coding RNA (ncRNA) and their mechanisms further investigated (Lee et al., 1993; Fire et al., 1998; Eddy, 2001), RNA biology came to the forefront (Todd and Karbstein, 2007). Along with advancements in experimental and transcriptomics techniques, which enabled a more detailed investigation of the translational control of cellular responses and phenotypes (Chan et al., 2010), interest in RNA modifications also grew, resulting in significant progress in the last 15 years. Recent discoveries, such as the first and second mRNA m^6^A demethylases FTO (Jia et al., 2011) and ALKBH5 (Zheng et al., 2013), as well as the identification of the METTL3/METTL14 methyltransferase complex (Liu et al., 2014), have triggered renewed interest in RNA modifications.

To date, a total of 163 post-transcriptional RNA modifications have been uncovered across all living organisms (Boccaletto et al., 2017) and are among the most evolutionarily conserved properties of RNAs (Li and Mason, 2014), revealing a “novel,” complex layer of biological regulation known as the *epitranscriptome* (Saletore et al., 2012). The functional diversity provided by these phenomena can indeed affect RNA structure, play a fundamental role in their interactions with other molecules and in regulatory networks, such as metabolic changes (Lewis et al., 2017), thus affecting every aspect of cellular physiology.

RNA modifications have been categorized as reversible and non-reversible. Among non-reversible modifications, we find well-studied phenomena such as RNA editing and pseudouridylation (Meier, 2011). Nonetheless, recent focus has shifted to *reversible* modifications, such as cytosine and adenosine methylations (Klungland et al., 2016). However, this classic distinction is being reassessed, in light of the discovery of “erasers” such as FTO and ALKBH5.

The importance of modifications in novel classes of ncRNA transcripts is also becoming relevant. Well-characterized chemical modifications in traditional classes of RNAs such as transfer (tRNAs) and ribosomal (rRNA) RNA, novel detection technologies and deep sequencing analysis (Veneziano et al., 2015, 2016), have paved the way for a fuller assessment of these molecular events also in regulatory ncRNAs, such as microRNA (Alarcon et al., 2015b) and long ncRNAs (Patil et al., 2016).

## RNA Methylation

**FIGURE 1:**
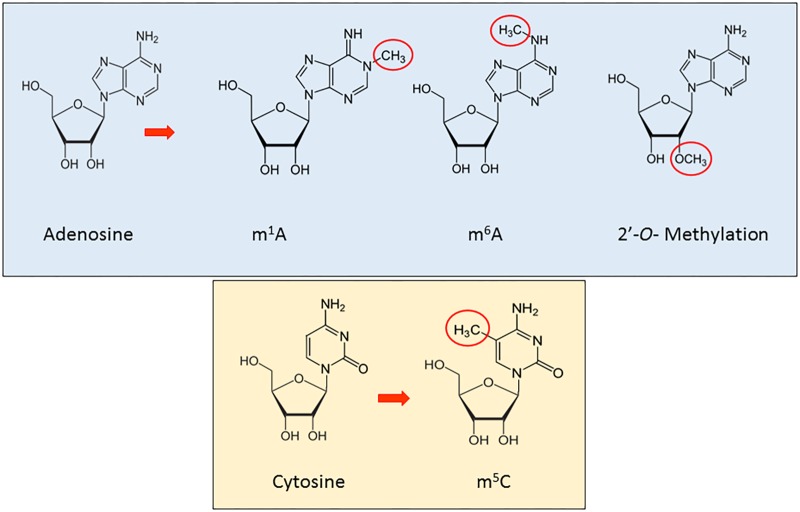
A schematic representation of the principal methylation modifications in eukaryotic RNA.

RNA methylation is a reversible, post-transcriptional RNA modification, affecting several biological processes, such as RNA stability and mRNA translation (Ji and Chen, 2012; Wang et al., 2014, 2015; Dev et al., 2017), through a variety of RNA methyltransferases, often using distinct catalytic strategies. Furthermore, recent studies have shown how the deregulation of proteins implicated in these modification phenomena is associated to disease (Supplementary Table 1A). In this section, we will review the main types and functions of methylation in ncRNAs (**Figure 1**).

### N^6^-Methyladenosine (m^6^A)

N^6^-methyladenosine (m^6^A) is the most abundant internal modification detected to date in mRNA (Roundtree et al., 2017). Discovered in the 1970s, its function has been thoroughly investigated only in the last decade (Rottman et al., 1974; Wang and He, 2014). This was driven by the recent discovery and characterization of evolutionarily conserved proteins able to encode (*writers*), decode (*readers*), and remove (*erasers*) methylation (Lewis et al., 2017). Since 1994, different writers have been identified, including METTL3 and METTL14, proven to regulate the circadian clock, differentiation of embryonic stem cells and primary miRNA processing (Dominissini et al., 2012; Wang et al., 2014; Alarcon et al., 2015b). These enzymes work in complex with proteins essential to the correct processing of RNA methylation (Schwartz et al., 2014): Wilms tumor 1-associated protein (WTAP), RNA-binding motif protein 15 (RBM15) and Protein virilizer homolog (KIAA1429). Additionally, the discovery of ALKBH5 and FTO has revealed the dynamic dimension of this modification phenomenon for cellular metabolism (Jia et al., 2011; Zheng et al., 2013). Recently, the YTH domain family proteins (YTHDF1–3) and YTH domain-containing protein 1 (YTHDC1) have been characterized as m^6^A readers, providing the first functional evidence of m^6^A (Wang et al., 2014).

The methyl group in m^6^A does not affect the Watson–Crick base-pairing (Liu and Jia, 2014), is highly conserved between human and mice and located in 5′ UTRs, 3′ UTRs, around stop codons, long internal and alternatively spliced exons (Dominissini et al., 2016; Li et al., 2016a; Lewis et al., 2017). It is also found in tRNA, rRNA, and small nuclear RNA (snRNA) as well as several long non-coding RNA, such as Xist (Dominissini et al., 2012). While not completely understood, m^6^A has been shown to play critical roles in the biological regulation of mRNA and ncRNA (Liu and Jia, 2014), particularly splicing, stability, turnover, nuclear export, and mediation of cap-independent translation (Meyer et al., 2015). Recently, Sun et al. (2016) have integrated all m^6^A sequencing data into a novel database, *RMBase*, identifying ∼200,000 N^6^-Methyladenosines (m^6^A) sites in human and mouse. Finally, Linder et al. (2015) mapped m6A and m6Am at single-nucleotide resolution and identified small nucleolar RNAs (snoRNAs) as a new class of m6A-containing non-coding RNAs (ncRNAs).

### N^1^-Methyladenosine (m^1^A)

Although the first studies on N^1^-methyladenosine (m^1^A) in total RNA date back more than 50 years (Dunn, 1961), only one study in the last decade has shed substantial light on function. m^1^A is a dynamic methylation event at the N^1^ position of adenosine, comprising the addition of a methyl group and a positive charge in the base, specifically in the Watson–Crick interface, obviously altering RNA-protein interaction and RNA secondary structures through electrostatic effects (Roundtree et al., 2017). m^1^A is abundant in tRNA and rRNA (El Yacoubi et al., 2012; Sharma et al., 2013) exercising major influence on structure and function (Anderson, 2005). Two groups recently found a strong conservation of the m^1^A pattern in several human and murine cell lines as well as in yeast, affirming the important role of this modification along the evolutionary chain. In particular, m^1^A has been shown to have a role in mRNA translation, *via* unique localization near the translation start site and first splice site (Dominissini et al., 2016; Li et al., 2016a; Roundtree et al., 2017) and by facilitating non-canonical binding of the exon–exon junction complex (Cenik et al., 2017).

### 2′-*O*-Methylation (2′OMe/Nm)

2′OMe is a very common RNA modification in abundant RNAs (rRNA, snRNA, tRNA) (Schibler and Perry, 1977; Borges and Martienssen, 2015; Roundtree et al., 2017) as well as in microRNA and it is fundamental for the biogenesis and function of these molecules (Ji and Chen, 2012). It was initially detected at the second and third nucleotide in many mRNA (Schibler and Perry, 1977). Further, it was observed that in rRNA, the loss of an individual modification had no apparent effect, while the deletion of 2–3 modifications in A and P site regions impairs translation and strongly delays pre-rRNA processing (Liang et al., 2009).

2′-*O*-methylation occurs in 3′ termini and is found to be important in plant biogenesis of small RNA, *inter alia* miRNA and siRNAs (Yu et al., 2005). Furthermore 2′-*O*-methylation plays an important role in protecting against 3′–5′ degradation and 3′ uridylation of some small RNAs as piRNAs in animals and Ago2-associated small RNAs in *Drosophila* (Ji and Chen, 2012). It has been found to be catalyzed by HUA-ENHANCER-1/piwi-methyltransferase (HEN1/piMET) enzyme.

### 5-Methylcytosine (m^5^C)

5-Methylcytosine (m^5^C) is an epitranscriptomic modification that involves the 5*^th^* carbon atom of cytosine as a target for methylation in poly(A) RNA, rRNA, tRNA, snRNA, and lncRNA (Amort et al., 2013, 2017; Lewis et al., 2017). While some of the proteins regulating m^5^C in different RNA have been identified, the biological function remains unclear (Nachtergaele and He, 2017). NOL1/NOP2/Sun domain family member 2 (NSUN2) together with DNA methyltransferase-like protein 2 (DNMT2) have been shown to be the writers of m^5^C, although to date no erasers or readers have been discovered (Lewis et al., 2017), though recently, investigators identified ALYREF as a potential reader of m^5^C (Yang et al., 2017). Several roles have been suggested for m^5^C, from the stabilizing of tRNA secondary structure and prevention of degradation or cleavage, to playing a role in translation when in rRNA and increasing the stability of mRNA transcripts (Esteller and Pandolfi, 2017).

## Methodologies for the Detection and Profiling of RNA Methylation

The recent advent of more sensitive and robust sequencing technologies (Li et al., 2016b), coupled with novel biochemical techniques (Song and Yi, 2017), has greatly improved the characterization and understanding of RNA modifications (Frye et al., 2016). This has allowed us to address challenges such as limitations with reverse transcription (RT) signatures and low transcript expression, as is the case with mRNA and lncRNA. Major advances in high-throughput sequencing methods (Helm and Motorin, 2017) have indeed allowed for the systematic identification of RNA modifications at single-nucleotide resolution, effectively distinguishing their distribution patterns in a transcriptome-wide manner.

Traditional biophysical targeted approaches for the detection and quantification of RNA modifications have further matured and provided the foundation for nearly all current high-throughput techniques (Vandivier and Gregory, 2017). Earlier methodologies relied on chromatography applied to direct sequencing, providing the very first evidence of modifications in RNA (Desrosiers et al., 1974). As these techniques only allowed detection of global patterns of modification, they were soon improved with the application of electrophoresis (Gupta and Randerath, 1979; Sprinzl and Vassilenko, 2005) and mass spectrometry (McCloskey and Nishimura, 1977; Kowalak et al., 1993) attaining for the first time base resolution. Recently other strategies, such as high-resolution melting (Golovina et al., 2014), have been implemented to narrow resolution. Nonetheless, an important strategy on which several high-throughput techniques were later developed, is based on the detection of variation in RT signatures (Brownlee and Cartwright, 1977; Motorin et al., 2007). As RNA modifications may interfere with the RT enzyme, inducing its arrest and/or the misincorporation of non-complementary deoxyribonucleoside triphosphates (dNTPs), this provided the foundation to several current methodologies exclusively RT-based as well as leveraging on chemical treatment of the RNA pool or the use of antibodies for the enrichment of modified RNA populations. Such is the case of techniques employing methyl RIP-seq (*MeRIP-seq*) (Mishima et al., 2015; Dominissini et al., 2016; Li et al., 2016a) coupled with various crosslinking techniques to improve the resolution window. For instance, in *m^1^A-ID-seq*, employ demethylases to generate a m^1^A-depleted control library for validation (Li et al., 2016a). In alternative techniques, such as *m^1^A-seq*, RNA pools undergo *Dimroth rearrangement* under alkaline conditions, converting m^1^A residues to m^6^A, thus producing different RT signatures that can validate the MeRIP data (Dominissini et al., 2016). Indeed, certain RNA modifications, such as m^6^A and m^5^C, are RT-silent. Despite simple antibody pulldown methods have satisfactorily mapped m^6^A sites (Dominissini et al., 2012; Meyer et al., 2012) and antibodies highly specific to methylated RNA bases have also been employed (Linder et al., 2015; Li et al., 2016a), most antibody-based methods do not provide nucleotide resolution. For this reason, more recent global approaches have paired antibody binding to covalent crosslinking at specific RNA sites, resulting in RT signatures able to improve resolution (Linder et al., 2015). For instance, after transcripts fragmentation in MeRIP protocols, antibodies forming non-covalent complexes with modified residues are further cross-linked to reactive residues nearby via UV light at distinct frequencies according to the specific techniques (i.e., *miCLIP* and *PA-m^6^A-seq*) for m^6^A detection (Chen K. et al., 2015). Such induced covalent crosslinks are then the sites at which RT stalls, yielding approximate or precise single-nucleotide resolution. Recently, an innovative detection technique has precisely elucidated m^6^A distributions across unknown regions *via* an antibody-independent strategy able to produce abortive cDNA signatures at m^6^A sites, greatly increasing resolution (Hong et al., 2018). In the case of m^5^C, *bisulfite sequencing* has yielded satisfactory results, although posing a few challenges. As unmodified cytosines are converted to inosines as a result of bisulfite treatment, m^5^C residues remain unaffected, providing a signature in cDNA. While this has been effective for highly abundant ncRNA populations (i.e., tRNA and rRNA) (Militello et al., 2014), degradation issues (due to higher pH conditions during treatment) and read mapping challenges have yielded poor results for low-abundance RNA species (Squires et al., 2012; Hussain et al., 2013; Jeltsch et al., 2017). An alternative approach termed “suicide enzyme trap” has been employed to characterize substrates of m^5^C-methyltransferases (m^5^C -MTases) NSUN2 and NSUN4 (Metodiev et al., 2014; Van Haute et al., 2016). By mutating m^5^C-MTases to form irreversible covalent bonds with target residues, the resulting stable enzyme–RNA complexes are suitable for immunoprecipitation and mapping. Such is also the case of the AZA-seq methodology formalized by Khoddami and Cairns (2014) in which “suicide inhibitor” nucleotide analog *5-azacytidine* is incorporated into cellular RNA and “traps” m5C-MTases for pulldown and sequencing.

Finally, 2′OMe too can be detected at base resolution *via* differential RT profiles, with or without chemical treatment. The *RiboMeth-seq* methodology (Birkedal et al., 2015; Krogh et al., 2016; Marchand et al., 2016, 2017) for instance, leverages on the ability of 2′OMe to preserve adjacent phosphodiester bonds from alkaline cleavage and produces a high-throughput coverage profile of under-represented positions at the extremes of reads. Nonetheless, chemical treatment is not strictly necessary. Indeed, earlier methods relied on the natural ability of 2′OMe to interrupt RT at low dNTP concentrations (Maden et al., 1995). Such principle was recently employed in the development of a high-throughput protocol proven to be more sensitive and specific than methods based on alkaline hydrolysis. These methodologies have specifically been assessed on 2′OMe modifications occurring in ribosomal and transfer RNA, while not as efficiently identifying such phenomena in low abundance RNA molecules such as mRNA and several ncRNAs. To address such deficiency, the recently published *Nm-seq* protocol leverages on the ability of 2′OMe to confer resistance to oxidation by sodium periodate to the ribose backbone of RNA molecules, thus allowing the enrichment and mapping of reads originating from RNA fragments whose internal 2′OMe have been exposed at the 3′ end *via* the elimination of non-modified nucleotides. Such technique has provided a sensitive and precise 2′OMe detection method for rare RNA classes (Dai et al., 2017).

Due to the time-consuming and labor-intensive nature of such techniques, many transcriptomes and potentially novel modifications remain unexplored. For this reason, computational methods have also been developed for the accurate evaluation of modifications events (Zhang et al., 2015; Liu et al., 2017). Moreover, given the error-prone nature of high-throughput techniques, it is strongly suggested that modification sites predicted from big data not be considered as candidates if not validated with at least one additional methodology (Helm and Motorin, 2017). All methodologies described above are summarized in Supplementary Table 1B.

## ncRNA Species and RNA Methylation: Functional Associations

### tRNA

tRNA methylations were first identified concurrently with the initial sequencing of the clover-shaped molecule (Holley et al., 1965a). Initially, it was suggested that such phenomena was probably the result of a network of diverse enzymes (Hurwitz et al., 1964). It is now clear that tRNA methylation is highly conserved and that tRNAs are the RNA class containing the majority of modified nucleosides among all discovered RNA species. With a total of more than 90 modified nucleosides identified (MODOMICS) (Boccaletto et al., 2017), all tRNA molecules from the three domains of life contain 13 methylated nucleosides out of 18 shared (Marck and Grosjean, 2002; Jackman and Alfonzo, 2013). Originally, it was thought that tRNA modifications in general were a straightforward, static process occurring on specific sites of distinct tRNA species. Given the recent characterization of major tRNA modification pathways, along with their associated tRNA methyltransferase enzyme families (Hori, 2014), a relevant diversity has emerged among living organisms. The presence of catalytic interactions, distinct RNA substrate recognition mechanisms and diverse chemical processes, all suggest a complex functional panorama. Generally, four functional categories can be attributed to tRNA methylation phenomena: preservation of secondary and tertiary structures (Helm and Attardi, 2004; Voigts-Hoffmann et al., 2007); thermodynamic stability (Yokoyama et al., 1987); protection from degradation and rapid tRNA decay (Kadaba et al., 2004; Alexandrov et al., 2006; Guy et al., 2014); translation control and fidelity (Anderson et al., 1998, 2000; Chan et al., 2010, 2012). It is thus evident that tRNA methylation contributes to RNA quality control systems, cellular localization (Kaneko et al., 2003), response to stress stimuli (Schaefer et al., 2010; Becker et al., 2012; Muller et al., 2013), proliferation and many other processes (Phizicky and Hopper, 2015). Most importantly, disruption of energy and amino acid metabolism pathways (i.e., depletion of methionine, necessary for methylation) can damage downstream the RNA modification system, resulting in partially modified tRNAs and thus translational errors (explaining why living organisms use the methionine codon as the initiation codon for protein synthesis) (Hori, 2014). Recently, researchers discovered the first tRNA demethylase, ALKBH1, as a novel post-transcriptional gene expression regulation mechanism (Liu et al., 2016). Finally, tRNA methylations and their enzymes may cooperate collectively in functional networks in order to support adaptive cellular responses (Chan et al., 2010; Tomikawa et al., 2010; Ishida et al., 2011).

### miRNAs

From transcription to decay, the multi-level process of the biogenesis of miRNAs is regulated by two main actors: processing enzymes such as DROSHA, DICER, and AGO proteins (Ha and Kim, 2014); and post-transcriptional modifications. Established RNA modifications, such as RNA editing events, have been shown to dynamically alter the sequence and/or the structure of miRNAs (Nigita et al., 2015; Nishikura, 2016) and consequently, in some cases, their function (Kawahara et al., 2007; Nigita et al., 2016). Recently, this has been also investigated in the context of miRNAs and RNA methylation.

2′OMe has been detected at the 3′-end of miRNAs (only in plants) and found to confer stability and protection from 3′-uridylation and degradation (Backes et al., 2012; Borges and Martienssen, 2015). m^6^A within 3′ UTRs has been generally associated with the presence of miRNA binding sites; roughly 2/3 of mRNAs containing an m^6^A site within their 3′ UTR also have at least one microRNA binding site (Meyer et al., 2012). In another study, Alarcon et al. (2015b) described how miRNAs can undergo N^6^-adenosine methylation (m^6^A) as a result of the intervention of METTL3 during pri-miRNA processing. The same authors also showed that m^6^A marks in pri-miRNAs allow for the RNA-binding protein DGCR8 to identify its specific substrates, promoting the beginning of miRNA biogenesis. Alarcon et al. (2015a) have further hypothesized that the RNA-binding protein HNRNPA2B1 could function as nuclear reader of the m^6^A mark, binding to m^6^A marks in pri-miRNAs, thus promoting pri-miRNA processing. Additionally, the effects of RNA *demethylation* on miRNA expression have also been investigated. Berulava et al. (2015) reported significant miRNA expression dysregulation as a result of knocking down m^6^A demethylase FTO, providing indirect evidence of co-transcriptional processing in the methylation of mRNAs and miRNAs. Finally, Chen T. et al. (2015) discovered that miRNAs positively regulate m^6^A installment on mRNAs *via* a sequence pairing mechanism. Methylation events in miRNAs add a new layer of complexity in the regulation of post-transcriptional gene expression and warrant future studies in order to fully elucidate the roles and functions of modified miRNAs.

### Long ncRNA

Although the majority of focus has been recently devoted to modifications in mRNA, 1000s of lncRNA transcripts have been detected containing a substantial number of modifications (Shafik et al., 2016). Evidence associating methylation with the most established lncRNA transcripts are just starting to be recognized. MALAT1 has been shown to bind with the m^6^A writer METTL16 at its 3′-triple-helical RNA stability element (Brown et al., 2016) specifically in its A-rich portion, after it was previously proven that MALAT1 can carry m^6^A (Liu et al., 2013). The presence of m^6^A has been further shown to destabilize the hairpin stems in the transcript, making them more flexible and solvent-accessible (Zhou et al., 2016) as well as more accessible for protein binding (Liu et al., 2015). Several putative m^5^C sites have also been detected in MALAT1 (Squires et al., 2012), but no enzymes have been identified. lncRNA HOTAIR (Khoddami and Cairns, 2013) possesses a specific m^5^C site which has been verified with a 100% modification rate (Amort et al., 2013). Finally, m^6^A events have been associated to XIST-mediated transcriptional repression (Patil et al., 2016) while m^5^C sites can prevent XIST-protein interactions, although it may not be a conserved mechanism (Amort et al., 2013). More detailed information can be found in Jacob et al. (2017).

## Author Contributions

GR wrote and set up the manuscript. GN and DV wrote and reviewed the content. SN-S supervised and reviewed the manuscript writing and development.

## Conflict of Interest Statement

The authors declare that the research was conducted in the absence of any commercial or financial relationships that could be construed as a potential conflict of interest.
